# Antiviral Potential of Algal Metabolites—A Comprehensive Review

**DOI:** 10.3390/md19020094

**Published:** 2021-02-06

**Authors:** António Pagarete, Ana Sofia Ramos, Pål Puntervoll, Michael J. Allen, Vítor Verdelho

**Affiliations:** 1 Edificio TecLabs, Faculdade de Ciências, Biosystems and Integrative Sciences Institute (BioISI), Universidade de Lisboa, Campus da FCUL, Campo Grande, 1749-016 Lisboa, Portugal; 2 Algae for Future, SA, Rua Eng. Clément Dumoulin Business Park, 2625-106 Póvoa de Santa Iria, Portugal; vitor.verdelho@gmail.com; 3 Institute of Biochemistry, University of Lübeck, Ratzeburger Allee 160, 23562 Lübeck, Germany; ramos.anasofiaferreira@gmail.com; 4 NORCE Norwegian Research Centre, Postboks 22 Nygårdstangen, 5838 Bergen, Norway; papu@norceresearch.no; 5 Plymouth Marine Laboratory, Plymouth PL1 3DH, UK; mija@pml.ac.uk; 6 College of Life and Environmental Sciences, University of Exeter, Exeter EX4 4QD, UK; 7 European Algae Biomass Association, Viale Belfiore, 10-50144 Florence, Italy

**Keywords:** algae, cyanobacteria, immunomodulatory effects, sulfated polysaccharides, lectins, HIV, coronaviruses

## Abstract

Historically, algae have stimulated significant economic interest particularly as a source of fertilizers, feeds, foods and pharmaceutical precursors. However, there is increasing interest in exploiting algal diversity for their antiviral potential. Here, we present an overview of 50-years of scientific and technological developments in the field of algae antivirals. After bibliometric analysis of 999 scientific references, a survey of 16 clinical trials and analysis of 84 patents, it was possible to identify the dominant algae, molecules and viruses that have been shaping and driving this promising field of research. A description of the most promising discoveries is presented according to molecule class. We observed a diverse range of algae and respective molecules displaying significant antiviral effects against an equally diverse range of viruses. Some natural algae molecules, like carrageenan, cyanovirin or griffithsin, are now considered prime reference molecules for their outstanding antiviral capacity. Crucially, while many algae antiviral applications have already reached successful commercialization, the large spectrum of algae antiviral capacities already identified suggests a strong potential for future expansion of this field.

## 1. Introduction

Viruses are the most abundant biological entities on Earth [1]. Their extreme diversity, often accompanied by very high mutation rates, continues to pose a threat to human society. Despite more than 99.99% of the viruses on our planet being harmless to humans or organisms related to human activities, a few viruses can have devastating effects particularly in intensive farming or areas of dense population. Viruses have diverse life cycles which are intricately integrated and in-tune with that of their hosts, which makes the discovery of efficient antiviral treatments an extremely demanding task. For example, viruses such as the human immunodeficiency virus [2], hepatitis C virus [3], dengue virus [4], herpesviruses [5], Ebola virus [6] or the most recent coronaviruses [7], can afflict a very high fraction of the world population. Despite extensive biomedical research efforts over the past half of a century, there are still no efficient vaccines against many of these viruses. Immunotherapeutics can be powerful but are often difficult and costly to develop, hence the interest in non-immunogenic alternatives. However, antiviral research has often been hindered by the side effects that a drug can have on its host organism and also by the common appearance of resistance to approved drugs [8]. At the beginning of the 21st century there were a mere ten licensed antiviral drugs. This number has been increasing as our capacity to understand the viral proliferation cycles has improved [9]. However, there is an obvious and constant need to research and discover new natural molecules that can be effective and well tolerated for treatment.

Algae and their extracts have numerous applications and have historically stimulated significant economic interest particularly as a source of fertilizer [10], feed [11] and food [12]. They comprise a very diverse group of organisms, not only in terms of their morphology, from microscopic unicellular organisms to seaweeds reaching 30 m in length but particularly in terms of the enormous diversity encoded in their genomes. A review done in 2010 by Yasuhara-Bell and Lu referred that *circa* 9% of all biomedical compounds were derived from algae [13]. Extensive research over the years on algal metabolites with pharmaceutical activity has established algae as a rich “mine” of molecules with anticancer, antitumor, antioxidant, anti-obesity, neuroprotective, antimicrobial, antinociceptive, anti-inflammatory and antiangiogenic activities [14]. It is not surprising that algae are also among the organisms regarded as potential sources of antiviral molecules.

Here we review what have been the main trends and achievements over the last half a century concerning the use and exploitation of algae-derived molecules displaying antiviral potential. An initial bibliometric analysis is followed by an in-depth look into the more promising molecules currently under study and use, including an overview of the most important clinical trials and patents.

## 2. Bibliometric Analysis

### 2.1. Progression Overtime

**Figure 1:**
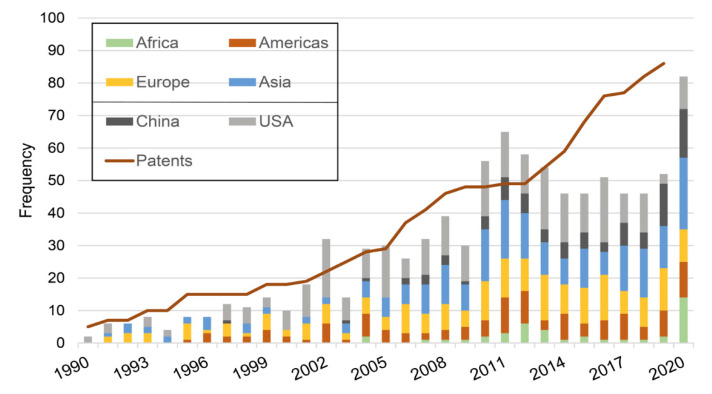
Progression of the number of publications and patents overtime. Histogram presents number of publications per year; the number of patents is presented in a cumulative line. Russia is included in Europe; “Americas” include all countries in the American continent except the USA; “Asia” includes all Asian and Oceania countries, except China.

Following curation, a total of 999 references were retained in our ‘algae antiviral’ database. The first reference dated back to 1958 and the last from December 2020. In total, we found publications from 58 different countries, which reflects the widespread geographical interest in the topic. Before the 1990s the number of publications per year was less than 5, with a steady increase observed through the 1990s onwards (Figure 1). Currently we witness a rate of *circa* 50 publications per year, although in the year 2020 alone counts a total of 86 publications. Until recently, the USA always had a lead role in the field. However, as in many other areas of research and economy, China has significantly increased its share in recent years. From a regional perspective, we observe that Europe, Asia and the Americas are balanced. In Europe, France and Spain are the leading countries. Africa and South America, which are the regions typically most affected economically and socially by the impact of viral diseases, are lagging in algae antiviral research. It is worthy of note that the data discussed here is based on declared author affiliations. Therefore, it does not reflect possible collaborations between countries but instead gives an idea of who is leading the research.

Before the 1980s, the antiviral activities of algae extracts were commonly tested in vitro. This very basic exploratory activity continues to this day, although few reviews on this methodology have been undertaken. Since the 1980s, a growing number of both in vitro and in vivo tests have been undertaken, as well as some pre-clinical trials and structural chemistry studies. During the last two decades, there has been a sharp increase in the number of publications and the diversity and complexity of studies. Along with the now common in vivo and in vitro testing, pre-clinical and clinical trials have become regular. Studies on transformation and expression of algae genes in plant or bacterial model systems are also accompanied by ‘omic approaches, such as genomics and transcriptomics. Computer-based molecule interaction modulations (studies not involving wet laboratory work) are now considered an important part of the research effort. The number of review articles on algae antivirals has also risen significantly.

### 2.2. Main Trends: Organisms/Molecules/Diseases

**Figure 2:**
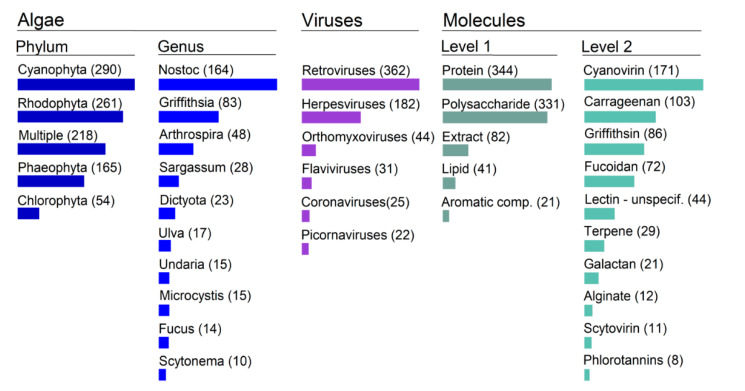
Ontological topography of the most meaningful categories in algae antiviral research. Bar length is proportional to the total number of publications per category.

Overall, there are 55% more publications on seaweed than on microalgae (Figure 2). Within the seaweeds, the reds (Rhodophyta) and the browns (Phaeophyta) have been more closely scrutinized than the greens (Chlorophyta). There are five times more studies on red than on green macroalgae. Among the microalgae, cyanobacteria have been the focus of more than 90% of the research on antivirals. At the genus level we found at least 81 different genera that were associated with putative antiviral activity. The most studied genus by a long distance is the cyanobacterial *Nostoc*, with the red seaweed Griffithsia second (accounting for 26% of all published studies between them). This is specifically due to the discovery of lectins Cyanovirin and Griffithsin (respectively), which are two of the most promising antiviral molecules found and which are also used as reference in a myriad of studies. Note that from the green macroalgae only *Ulva* makes it to do the top 10 of most studied genera.

There has been an exploration of algae metabolite activity against a broad range of viruses, encompassing a total of 28 different viral families and 61 different viruses. However, the distribution is extremely skewed towards Retro and Herpes viruses, which take the very large majority of the research effort (Figure 2). For example, there are 10 times more studies on HIV than on the Influenza orthomyxovirus (which is 3rd on the list). It is worthy of note that prior to 2020, coronaviruses had hardly been the focus of algae antiviral activity studies.

A broad range of molecules have been tested; we counted 37 different types in our assembled library. Most of the attention has gone to proteins (particularly lectins) and sulphated polysaccharides (Figure 2). Here also the distribution is also particularly skewed, with a small number of molecules receiving the bulk of the attention. The extreme example of that is the lectin protein Cyanovirin, isolated from the cyanobacteria *Nostoc*. Since its discovery in 1997 by Boyd and colleagues, this molecule has already been the subject of more than 160 research works. It is considered a major reference in with regards to molecules displaying anti-HIV effects.

**Figure 3:**
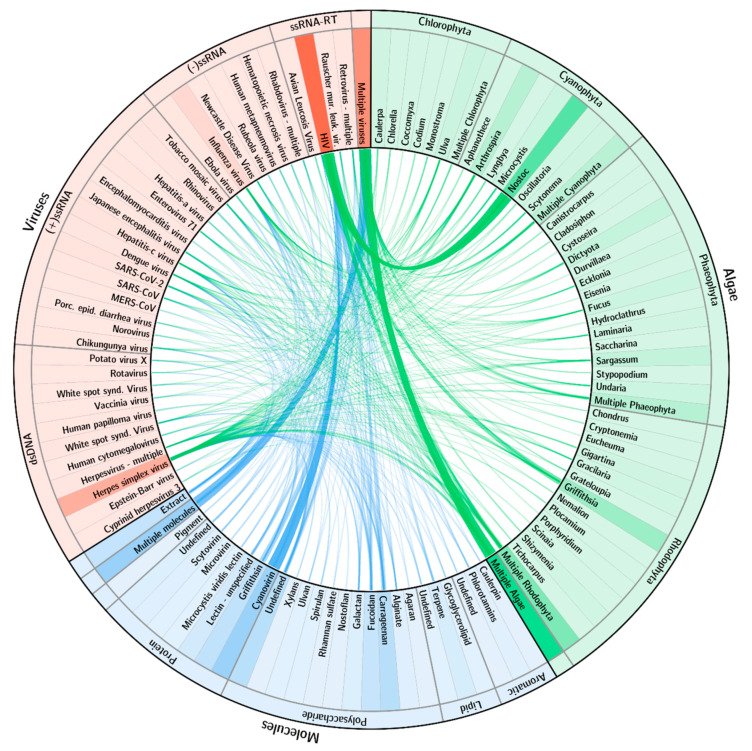
Research interactions among algae, molecules and viruses. Algae/viruses shown in green. Molecule/viruses shown in blue. Width of each link is proportional to the number of references on those two respective items. Background color intensity of each item is proportional to the number of research references it has. Only items with 2 or more references are presented.

The scientific community has explored numerous combinations of interactions between types of algae, molecules and viruses (Figure 3). As expected, research has been extremely skewed towards some particular, well-proven interactions. The proteins cyanovirin (mainly) and griffithsin have been the most studied for applications against the retrovirus HIV. As a result, the cyanobacteria *Nostoc* and the red alga *Griffithsia* also have strong interactions with that virus. Research on herpesviruses has also been relevant but resulting from a wider spread of both algae and respective molecules. A very significant number of studies explores the potential impact of multiple algae or molecules against various viruses.

### 2.3. Aquaculture and Agriculture

**Figure 4:**
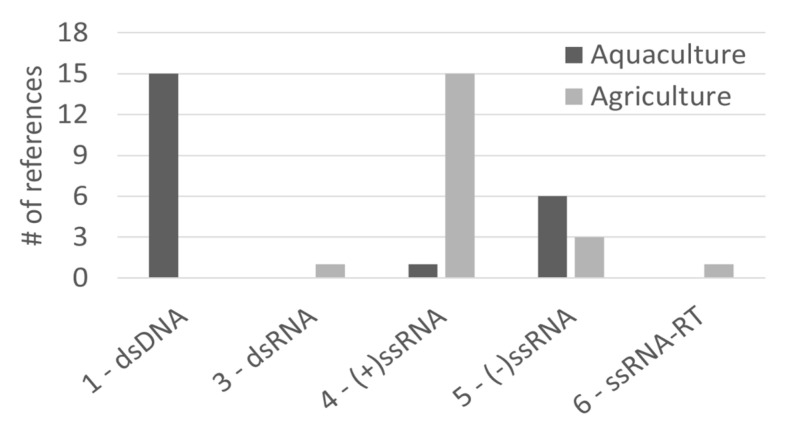
Number of publications that refer to agriculture or aquaculture research, discriminated per viral Baltimore group.

A rather small number of studies have been published on the antiviral potential of algae in agriculture (26 studies, Impact Factor 2.9) and aquaculture (27 studies, Impact Factor 2.9) settings. The algal species types used in the two fields is also similar, yet we could only find microalgae reports for uses in aquaculture. Regarding viral types, different patterns were observed for agri- and aquaculture, with an agriculture focus on group (+)ssRNA viruses, while aquaculture research focuses more on dsDNA viruses (Figure 4), potentially hinting at a difference in economic impact for virus types in aquatic and terrestrial settings. It is possible to predict a large growth potential for algae antivirals targeting agricultural crops, promoted by the increase of intensive mono-specific agriculture practices.

## 3. Polysaccharides

Polysaccharides are the most studied algal polymers from an antiviral activity perspective. They have often been described as having promising and in a few cases confirmed, therapeutic applications when used either alone or associated with existing antiviral drugs [15,16]. Polysaccharides can be extracted from the walls of any alga, from which they usually account for more than 50% of its composition. Despite their extensive structural diversity, most of these “antiviral” polymers are negatively charged and present a high sulfation level. Crucially they often combine a potent antiviral effect with low toxicity, making them interesting solutions for limiting viral infections in clinical contexts. Polysaccharides from algae have shown important antiviral effect against a broad spectrum of human and non-human pathogens. Below we address the most relevant discoveries and antiviral applications for algae-derived polysaccharides, focusing notably on carrageenans, fucoidans, ulvans and spirulan.

### 3.1. Fucoidans

Fucoidans were first discovered in 1913 [17]. They are a very diverse group of sulphated polysaccharides derived from the cell walls of brown seaweeds. Their heteropolysaccharide structure is composed of L-fucose and sulphate groups. Other monosaccharides including uronic acid, galactose, xylose, mannose, rhamnose, glucose, arabinose and xylose can also be present [17]. Each fucoidan isolated from a different alga species is a different molecule with unique structural elements, which leads to a wide range of biological activities [18]. Given that they are non-toxic and have strong potential in many therapeutic applications, fucoidans have been extensively studied [19]. In our working database of selected algae antiviral related papers, we could identify 130 scientific articles that had the term “Fucoidan” in the title or the keywords.

Fucoidans have shown in vitro antiviral activity against HIV [20,21,22], HsV [23,24,25,26,27,28], Influenza virus [29,30], human cytomegalovirus [23,24], bovine viral diarrhea virus [31,32,33] and murine norovirus [34]. They prevent viral entry by competing for the attachment to positively charged envelope glycoproteins, in a process that is related to the level of sulphate groups present in the fucoidan [35]. It has also been shown that pre-incubation of influenza virus with fucoidan, prior to inoculation onto Madin-Darby Canine Kidney (MCDK) cells, significantly reduced the number of plaques. This suggests that the fucoidan can potentially inactivate the virus by direct contact [36]. Recently, in vitro tests with two fucoidans extracted from the edible brown algae showed the molecules to be potent inhibitors of SARS-CoV-2 [37]. Crucially, fucoidans clearly outcompeted Remdesivir, which is currently approved for emergency use for severe COVID-19 infections [38].

Fucoidans have been tested in vivo mice models for their activity against different viruses (Table 1). Orally administered fucoidan from *Undaria pinnatifida* decreased Avian Influenza A virus (subtypes H5N3 and H7N2) replication, while augmenting the production of antibodies [39]. In another study the oral administration of the same fucoidan onto immunocompetent and immunocompromised mice, infected with a lethal dose of influenza virus, reduced virus replication, weight loss and mortality in both groups, while increasing their life span [40]. More interesting was that the fucoidan did not lead to the appearance of drug-resistant mice, which is common when treating with the standard antiviral oseltamivir [40]. The same result was found for an intranasal application of the fucoidan extracted from *Kjelmaniella crassifolia* [36]. Intraperitoneal administration (10 mg/kg) of fucoidans from *Fucus evanescens* protected mice with *circa* 50% efficacy from lethal intravaginal Herpes simplex-2 infection [28]. Further pharmacokinetic information on antiviral properties of fucoidans can be found in the recent review by Shikov and his colleagues [41].

### 3.2. Carrageenans

Carrageenans are a diverse group of polysaccharides present in the cell walls of numerous red seaweed species (Rhodophyta) [53]. Extraction of the hydrophilic colloids from red algae is at least as old as 1810, when they were being retrieved from algae collected on the coasts of Ireland [54]. Carrageenans are widely used in the food industry but also in pharmaceutical, cosmetic, printing and textile formulations [55]. They are the most studied of the algae-derived molecules, in part due to their therapeutic effects.

In our working database of selected algae antiviral related papers, we could identify 160 scientific articles that had the term “Carrageenan” in the title or the keywords. Carrageenans are composed of repeating disaccharide units of D-galactose. Like many other algae-derived polysaccharides, carrageenans are also sulphated to different extents, which contributes to their structural and metabolic diversity and is related to their antiviral capacities [35].

Carrageenans tend to prevent the physical attachment and entry of the viral particles [56,57,58]. As expected, this mechanism varies according to the type of carrageenan, viral serotype and host cell type [35,59]. Since 1987, carrageenan strong antiviral effects have been demonstrated in vitro for a panoply of very problematic enveloped and non-enveloped viruses. This includes HsV [60,61,62], HIV [63], Hepatitis-A [59], Human papilloma viruses [64], Dengue virus [57,65,66], Rhinoviruses [67], Japanese encephalitis virus [58] and Tobacco mosaic virus [68]. For HsV-2, for example, carrageenan extracted from *Meristiella gelidium* has demonstrated a selectivity index of 25,000, clearly indicating it should be further evaluated for treatment against that virus [65]. In vivo it displayed potent inhibitory effects against HsV [69,70] and Murine cytomegalovirus [71]. Carrageenans antiviral capacities have been evaluated and demonstrated in different clinical trials (see section below).

### 3.3. Ulvans

Ulvan is an abundant cell wall polysaccharide found in species of the green seaweed genus *Ulva,* reaching up to 36% of the alga’s dry weight [72]. Its repeating disaccharide structure comprised of an uronic acid linked to a sulphated neutral sugar is a candidate for the modulation of processes and functions carried out by mammalian polysaccharides [72]. Antiviral capacities are widespread among many Ulvans from diverse *Ulva* species (e.g., *U. compressa* [73], *U. lactuca* [74], *U. clathrata* [75], *U. intestinalis* [76], *U. armoricana* [77] or *U. pertusa* [78]). These ulvans show antiviral activity against a panoply of enveloped viruses, notably HsV [73,77], Newcastle disease virus (NDV) [75], Japanese encephalitis virus (JEV) [74], Dengue virus [79], Influenza (H1N1) [29], Avian influenza virus [78], Vesicular stomatitis virus [80] and Measles virus [76].

Regarding the avian flu AIV-H9N2, ulvan from *U. pertusa* only had a moderate antiviral activity on its own (circa 40% viral inhibition) [78]. However, when combined with a vaccine against that same virus, it led to a ~100% increase in antibody titer relative to the vaccination alone. It was suggested that the immunomodulatory effects of the ulvan were responsible for that enhancement of the humoral immune response [78]. Another success case was the 100% HsV inhibition achieved with a highly sulphated (SO_3_^−^ = 22%) ulvan fraction from *U. compressa* [73]. Ulvans may also be of important help controlling viruses in poultry related activities, such as the Newcastle Disease Virus, a fatal virus found in chickens that can cause significant economic losses. In vitro tests using Vero cells showed that ulvan can inhibit viral entry with an IC_50_ of 0.1 μg/mL [75]. Despite these very promising results we did not find reports of in vivo antiviral trials with Ulvans.

### 3.4. Spirulan

Spirulan, existing as an ionic form (calcium or sodium), is a sulphated polysaccharide isolated from *Arthrospira platensis* (commercially know as Spirulina) [81]. Along this study it was also considered that until recently *Arthrospira platensis* was referred as *Spirulina platensis*. Since its discovery spirulan has proved particularly effective against enveloped viruses, including HsV-1, mumps virus, measles virus, human cytomegalovirus, influenza A virus and HiV-1 [82]. It selectively inhibits the penetration of the viruses into the host cells and is more powerful than dextran sulphate. That same study revealed that Calcium spirulan is ineffective against the non-enveloped viruses Poliovirus and Coxsackievirus [82]. Another in vitro assessment of spirulan antiviral activities, using direct virus-specific approaches, showed strong inhibition of human cytomegalovirus, HsV-1, HsV-6 and HiV-1 [83]. The effects on herpesviruses were more pronounced when the cells were treated prior to the viral inoculation, which indicates a mode of action that prevents viral entry [83,84]. A recent study has demonstrated the great potential of this substance against herpesviruses both in vitro and in a clinical trial; Calcium spirulan blocks the attachment and penetration of HsV-1 into mammalian epithelial cells with a potency at least comparable to that of acyclovir. It also inhibited entry of Kaposi sarcoma–associated herpesvirus/HsV-8 [85].

## 4. Lectins

Lectins are one of the most interesting class of molecules extracted from algae. These diverse peptides are characterized by having highly specific carbohydrate-binding domains. Lectins are found in all types of algae and several have been identified that show potential for the development of new drugs [86]. Due to their capacity to bind with high specificity to outer membrane carbohydrates; lectins predominantly show antibacterial, antifungal and antiviral activities. The latter mostly by interacting with viral envelope glycoproteins [87]. We address in detail the work that has been done on the most promising algae-derived lectins and which have been the subject of extensive research due to their extraordinary potential as antiviral application. Further information on algae lectins and their therapeutic effects can also be found elsewhere [88,89,90,91,92].

### 4.1. Griffithsin

Griffithsin was first isolated from an aqueous extract of *Griffithsia sp.*, a red seaweed [93]. The Mori study revealed a protein that had very low homology with other known proteins and a very strong capacity to inhibit HiV-1 in vitro (EC_50_ range 0.043–0.63 nM) [93]. Since then, this protein has been extensively studied for its anti-HIV capacity but also against many other viruses. In our search we could identify 123 scientific articles that had the term “Griffithsin” in the title or the keywords. Regarding HIV, the *Griffithsia* lectin is still considered one of the most potent HIV entry inhibitors know to date. In fact, Griffithsin is more potent than broadly neutralizing antibodies (bNAbs), including the high-mannose-binding 2G12. In vitro and in vivo (Table 1) trials have demonstrated that Griffithsin is not only a highly potent HIV entry inhibitor [94] but it also improves the antibody responses [95,96] and prevents cell fusion and cell-to-cell transmission of HIV [97]. Mucosal delivery of Griffithsin, via a silk fibroin biomaterial vehicle, resulted in significant protection against HIV in human cervical and colorectal tissue, as well as against SHIV infection in macaque vaginal and rectal tissues [98].

Griffithsin antiviral capacity has also been extensively demonstrated in vivo against many other enveloped viruses, such as Hepatitis C virus [99], Herpes simplex virus [46], Japanese encephalitis virus [44], Nipah virus [100], Hantaviruses [101] and coronaviruses MERS-CoV [102] and SARS-CoV [43,103]. Regarding the latter, a 100% survival rate was registered in treated mice (5 mg/kg dose intranasally delivered 4 h before infection) that had been infected with a viral dose known to cause at least 75% mortality [43]. Also, Human papillomaviruses, that are not enveloped, are inactivated by Griffithsin through a glycosylation-independent process [47].

Recombinant Griffithsin is now produced in *Escherichia coli* [104] and, in larger quantities, in the plant *Nicotiana benthamiana* [105], while trials in other systems progress [106,107]. Considerable research efforts are taking place to develop new/optimized strategies of Griffithsin administration, such as polylactic acid nanoparticles for vaginal delivery [108], pH-responsive delivery from electrospun fibers [109], fast-dissolving inserts [110] and gels for topical application [111].

### 4.2. Cyanovirin-n

Cyanovirin-n (CV-N) is the most studied algal lectin. We could identify 365 scientific articles that had the term “Cyanovirin” in the title or the keywords. This lectin was discovered in the cyanobacteria *Nostoc ellipsosporum* [112]. Cyanovirin consists of a 101-aa long polypeptide, including four cysteine residues that form two intra-chain di-sulphide bonds. These bonds are fundamental as they stabilize the protein’s structure and determine its antiviral activity [113]. Initial interest in CV-N relied on its exceptional anti-HIV capacity [114]. By strongly interacting with HIV’s envelope protein gp120, it prevents the virus binding to the host CD4 T-cell receptor and the chemokine CCR5 and CXCR4 co-receptors [115,116]. Further details on the CV-N structure and the role each domain plays in the interaction with virus and host cells can be found in existing literature [113,117,118,119]. CV-N has been mostly studied for its anti-HIV activity but it has also demonstrated in vitro to be a potent inhibitor of other enveloped virus, notably Herpes simplex virus [115], Influenza virus [120], Measles virus [115] and Ebola virus [121].

The broad spectrum of antiviral activities, along with very specific oligosaccharide targeting, confers CV-N strong potential for the prevention of sexually transmitted diseases. Female macaques (*Macaca fascicularis*) treated with CV-N topical gel were resistant to a chimera of HIV viruses [49]. A 63% reduction of HIV transmission was registered when macaques were dosed vaginally with a Lactobacillus expressing CV-N [50] and it has proven to be a preventive measure in rectal transmission [48] (Table 1). Possible CV-N side effects observed in in vitro testes with peripheral blood mononuclear cells, such as cell activation, mitogenicity and increased cytokine production, have conditioned a more determined approach to the development of CV-N as a microbicidal anti-HIV agent [122]. Notwithstanding, extensive research has been conducted in two ways to produce CV-N in the quantities and at the costs required for a broad utilization, including recombinant expression in different bacterial hosts (e.g., *Escherichia coli* [123,124], *Lactococcus sp.* [125], *Lactobacillus sp.* [126]) and transgenic plants [127]. In the case of expression in *Nicotiana tabacum*, for example, a yield of 130 mg of CV-N per g of fresh leaf tissue can be obtained [128]. A way to increase the activity of CV-N is to blend it with the plant-derived antibody mAb b12 that neutralizes HIV [129]. A recent study as also showed how an extended CV-N with a Gly4Ser linker is more efficient inhibiting HIV [130].

### 4.3. Scytovirin

Scytovirin (SVN) is a 95 amino-acid long protein isolated from the cyanobacterium *Scytonema varium* that revealed a potent anti-HIV activity following its discovery [131]. Other studies have then demonstrated its strong capacity to inhibit HIV replication in vitro [132,133]. It is, however, less potent against that virus than Griffithsin or CV-N, revealed by significantly higher IC_50_ values, on the order 10s of nM [132]. It has on the other hand demonstrated high affinity to bind to mannose-rich oligosaccharides on the envelope glycoprotein (GP) of a number of other viruses, blocking the entry into target cells. A good example is SVN’s capacity in vitro to inhibit the replication of hepatitis c virus [134]. Even more relevant is its rare capacity to inhibit the highly pathogenic Zaire Ebola virus. In vitro it has been shown to inhibit Ebola virus with an EC_50_ concentration of 50 nM (significantly lower than CV-N) and also capable of preventing the replication of the Angola strain of the related Marburg virus (MARV), with a similar EC_50_. In vivo the SVN treatment prevented the death of most Ebola virus-infected mice, when while all infected, untreated mice died [51] (Table 1). Molecular dynamics simulations of the interactions of SVN with the single-stranded RNA Dengue virus [135] places this protein also as one of the best candidates to use against this devastating Flavivirus, which is responsible for circa 400 million human infections per year [136].

Like other algal lectins, SVN is only produced at concentrations in the ng/L region in the native organism, *Scytonema varium*. More efficient production systems are necessary if this molecule is to be used as a microbicide tool. To answer that challenge, Xiong and colleagues successfully expressed recombinant SVN in *Escherichia coli*, reaching concentrations of 10 mg/L while maintaining its anti-HIV capacity [137]. More recently a bioengineered intravaginal isolate of *Lactobacillus plantarum* that expresses SVN successfully demonstrated anti-HIV activity [138].

### 4.4. Microvirin

Recently, a new algal lectin protein with great potential has been discovered, namely Microvirin isolated from *Microcystis aeruginosa* cyanobacteria [139]. This simpler 108 aa-long lectin is a monomer in solution, with a single glycan-binding site that also recognizes terminal α1,2- mannose sugars [140]. It has been shown to neutralize in low nanomolar concentrations a wide range of HiV-1 strains [139]. Although being similar to CV-N, it presents a simpler structure and does not exhibit the toxicity or mitogenicity displayed by CV-N [139]. Therein resides its strong potential to become a widely used microbicide. The less elaborate MVN structure, containing only a single glycan-binding site and a simpler footprint of gp120 glycan binding [140], has recently allowed the creation of a bifunctional chimera with dual action virus entry inhibition that is more efficient against HIV-1 than CV-N [141]. Also, strong potential relies on oligomeric engineered MVN, which shows an enhanced capacity against Hepatitis C virus [142].

## 5. Diterpenes

Diterpenes are another class of structurally diverse natural products, which as the name implies are composed of two terpene units. Terpenoids are widely found in marine organisms and many are promising drug candidates (or precursors) due to their remarkable pharmacological activity. During the last 20 years there has been an important increase in research related to diterpenes extracted from algae which exhibit antiviral properties. These studies have focused almost entirely on the brown algae order Dictyotales and more particularly on species of the genus *Dictyota* [143]. Several in vitro tests have shown that diterpenes from *Dictyota* seaweed are very effective against HIV [144]. These include diterpenes from *D. menstrualis* [145,146,147], *D. pfaffii* [148,149], *D. friabilis* [150] and *D. plectens* [151]. The later, for example, shows significant inhibitory activity against HiV-1 replication in human embryonic kidney 293T cells with IC_50_ values ranging between 16.1–30.5 μM. In another genus, diterpenes from the species *Stypopodium zonale* (Dictyotales) also show a strong effect against HIV [152].

Another virus that has been thoroughly studied is the Herpes virus HsV-1. *Dictyota pfaffii* [153], *D. menstrualis* [154] and *Canistrocarpus cervicornis* (Dictyotales) [155] have all shown to strongly inhibit HsV-1 infection in Vero cells. The latter has also been tested in vivo on mice infected with HsV-1 (Table 1). The untreated animals showed significantly more severe lesions than the ones treated with a 2% diterpene extract ointment (*p* < 0.05) or acyclovir (*p* < 0,01). These results, combined with the absence of secondary effects, suggest that *C. cervicornis* extract is a very promising anti-HsV agent for cutaneous use [52]. *D. plectens* diterpenes also showed very good results against the highly pathogenic Asian Avian Influenza A (H5N1) virus, with inhibition rates of 50–62% at 30.0 μM [151].

Another important virus against which Dictyota diterpenes have showed good effect is the Zika virus [156]. This virus has become a major concern of public health in recent years due to its widespread dissemination, particularly in the American continent. So far there are no vaccines available and effective drugs against Zika infection are also missing. A recent and very promising study with *D. menstrualis* extract has showed that its cyclic diterpenes can inhibit Zika virus replication by more than 74%. Mechanisms of action of these diterpenes vary between virucidal potential and inhibition of viral adsorption. What is more interesting is that, when associated with the antiviral Ribavirin at suboptimal dosages, they can completely inhibit Zika viral replication [157].

## 6. Clinical Trials

A total of 16 clinical trials on the applicability of algae-derived antivirals were found (Table 2). A large proportion of these trials (63%) were trying to find a viable treatment against HIV. Seven of those studies, all lead by the Population Council (USA), attempted to use carrageenan against that disease. While all applications were shown to be well accepted by the body, the treatments failed to prove efficacious. More recently, attention has turned to the protein Griffithsin, with a couple of trials showing promising results. Other relevant sexually transmitted viruses that were the focus of human trials are Papillomaviruses (3 trials). The application of Carrageenan-based formulations to prevent the spread of these viruses also seems promising.

Carrageenans are the algae-derived molecules whose antiviral activity has been most tested in human trials (Table 2). Such studies have focused on the sexually transmitted viruses HIV, HsV and HpV, but also against rhinoviruses. The reported capacity of iota-carrageenan to interfere directly with adsorption of HpV to human sperm cells has led to two trials that have so far suggested that carrageenan-based gel is effective against transmission of that virus [158] and also that it is well tolerated [159]. A third trial coordinated by the McGill University (Canada) is currently on-going [160]. Since 1997, seven clinical trials have attempted to demonstrate the efficacy of carrageenan-based gel (carraguard) as vaginal microbicide against HIV and HsV transmission. However, none of those trials was able to clearly prove the efficacy of this topical application (Table 2). The most successful case of carrageenan antiviral utilization are the nasal-spray applications that have been developed against rhinoviruses. Two clinical trials have clearly demonstrated that direct local administration of carrageenan with nasal sprays reduced the duration of rhinovirus-associated cold symptoms (Table 2). These extraordinary results led to the approval by the Joint Expert Committee on Food Additives (JECFA) of carrageenan as safe for medical purposes, patent registration and commercialization (Coldamaris) by Marinomed Biotechnologie GmbH (Austria). To the best of our knowledge, carrageenans are the only algae-derived compounds that have passed all clinical trials and are commercialized for their clear antiviral properties, notably against common cold symptoms and associated viruses [161].

A recent study has demonstrated the great potential of Calcium spirulan against herpesviruses [85]. That clinical trial performed on 198 volunteers showed that the prophylactic effect of a Calcium spirulan and microalgae extract containing cream against Herpes labialis was superior to that of acyclovir cream (Table 2).

Two clinical trials have been launched to evaluate the safety and efficiency of using Griffithsin gel as prophylactic measure against HIV transmission (Table 2). The first study, led by the Population council, reported that Griffithsin administered vaginally for 14 Days was well-tolerated, with anti-HIV activity up to 8 h post administration [162]. The other current study on-going, named PREVENT (pre-exposure prevention of viral entry) aims at providing comprehensive data (e.g., number and frequency of adverse events, concentration in the blood and changes in humoral antibody response) to allow an informed decision on whether to proceed the efforts to use Griffithsin as a topical microbicide [163].

## 7. Patent Analysis

**Figure 5:**
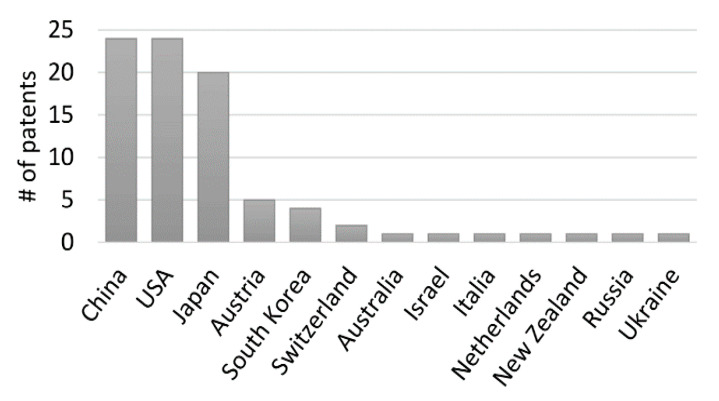
Number of patents per country. Predominance of China and USA. Japan, with 20 patents, is also an important player in the field.

The first patent protecting IP ideas on the utilization of algae-derived substances appeared in Japan in 1981 and since then has been steadily increasing to reach a total of 84 patents found nowadays on Espacenet database (Figure 1). The inventors behind these patents came from a limited number of 13 countries (Figure 5). This technology protection efforts were clearly led by China and the USA (each with 24 patents) and the forerunner Japan (20 patents). Most of the patents (44) claim to protect a method of antiviral production. This is done by describing a method of extraction of the particular extract or molecule, describing its antiviral effect against a particular virus or a group of viruses, and/or by presenting the detailed composition of a compound, including algae-derived substances, with antiviral potential. A considerable number of patents (37) protect not only the method for antiviral production but its method of application as well. The remaining five patents focus only on a method for antiviral use. In what regards applications, the vast majority of the patents (79%) relate to potential benefits for human health, either via pharmaceutical usage or as food supplements. Aquaculture and animal husbandry (13 and 12 patents each, respectively) have also been the subject of important intents of technology protection in this field.

All the major classes of algae (Rhodophyta, Phaeophyta, Chlorophyta and Cyanobacteria) are well represented in terms of patents. Singularly, it is the cyanobacteria Nostoc, with 12 patents, that stands out as the species most protected for its technological potential in antiviral applications. In terms of molecules, most patents rely on the use of algae polysaccharides, notably carrageenan. We also found 20 patents on algae proteins, from which Cyanovirin (9) and Griffithsin (5) are predominant. Worthy of note are the significant number of patents (18) protecting a “direct” usage of algal extracts in antiviral applications. Regarding the viruses, the identified patents claim to have effective applications against more than 40 different viral genera. The majority focus on HIV, Influenza or Herpesviruses. The general category “Aquaculture viruses” was also well represented.

## 8. Materials and Methods

In January 2021 a comprehensive search of literature was conducted in the following databases: PubMed^®^ (US National Library of Medicine, USA), ScienceDirect^®^ (Elsevier Properties S.A, USA), Scopus^®^ (Elsevier Properties S.A, USA) and Web of Science^®^ (Clarivate Analytics, USA). The following keywords strings were used to search all fields: “algae AND antiviral”; “microalgae AND antiviral”; “algae AND anti-viral”; “microalgae AND anti-viral”; “algae AND antiretroviral”; “microalgae AND antiretroviral”; “spirulina AND virus”; “seaweed AND antiviral NOT Algae NOT microalgae”; “cyanobacteria AND antiviral NOT Algae NOT microalgae”; “spirulina AND antiviral NOT Algae NOT microalgae”; “diatom AND antiviral NOT Algae NOT microalgae”; “cyanovirin AND virus”; “cyanovirin AND antiviral NOT Algae NOT microalgae”; “galactan AND antiviral NOT Algae NOT microalgae”; “carrageenan AND antiviral NOT Algae NOT microalgae”; “fucoidan AND antiviral NOT Algae NOT microalgae”; “spirulan AND antiviral NOT Algae NOT microalgae”; “alginate AND antiviral NOT Algae NOT microalgae”; “lectin AND alga AND virus”; “Griffithsin AND antiviral NOT Algae NOT microalgae”; “Phycocyanin AND antiviral NOT Algae NOT microalgae”; “Phlorotannins AND antiviral NOT Algae NOT microalgae”; “Ambigol AND antiviral NOT Algae NOT microalgae”; “Caulerpa AND antiviral NOT Algae NOT microalgae”; “Fucus AND antiviral NOT Algae NOT microalgae”; “Ulva AND antiviral NOT Algae NOT microalgae”; “Lyngbya AND antiviral NOT Algae NOT microalgae”; “Microcystis AND antiviral NOT Algae NOT microalgae”; “Ascophyllum AND antiviral NOT Algae NOT microalgae”; “Red Alga” AND antiviral NOT Algae NOT microalgae”; “rhodophyta AND antiviral NOT Algae NOT microalgae”; “syndecans AND algae”; “antiherpetic AND alga”; “antiherpetic AND microalga”; “anti-herpetic AND alga”; “anti-herpetic AND microalga”; “hsv AND alga”; “hsv AND microalga”; “coronavirus AND alga”; “COVID AND alga”; “Scytovirin”; “cyanovirin”; “griffithsin,” Studies that involved an algae-derived substance that was being studied/developed for antiviral purposes (even if being produced in a third recombinant organism) were kept. Studies that addressed the potential use of algae-derived molecules for other medical purposes (e.g., antimicrobial), even if the study used a molecule or alga with proved antiviral effects, were discarded.

The hits obtained were imported into an Endnote library and duplicate articles were automatically removed using the respective program function. The remaining references were manually screened to further exclude missed duplicates and articles that did not follow under the scope of this review. Articles were retained only when there was a clear connection with algae and antivirals. For example, studies on bacteria-expressed genes of clear algae origin, such as cyanovirin, were retained.

In December 2020, a search for clinical trials with algae-based antivirals was performed on the databases ClinicalTrials.gov from the U.S. National Library of Medicine (www.clinicaltrials.gov) and the International Standard Randomized Controlled Trial Number (www.isrctn.com).

In August 2020, based on the results from the literature analysis, a search for patents was conducted to identify the main technological trends in field of antivirals from algae. Patent numbers were collected from Espacenet database (worldwide.espacenet.com) using a variety of search options. The ten most relevant algae species or molecules were searched in combination with the word “antiviral” in the title, abstract or claims. The hits obtained were then manually curated and tagged according to year of earliest priority, algae types, molecule types, viral types, type of patent, type of application and country.

## 9. Conclusions

Algae antiviral capacity has been explored for a wide portfolio of health conditions. Nonetheless, it is notable that the vast majority of research investment has focused on HIV and secondarily on Herpesviruses. All classes of algae have been shown to display great antiviral potential. The translation from potential to potent antiviral production platforms is dependent on a plethora of factors, dominated primarily by an ability to boost cellular production through environmental stimuli or by recombinant production in established industrial platforms.

Commercial success of extracted molecules has been dominated by the groups, Rhodophyta and cyanobacteria. Among many very promising sulphated polysaccharides, carrageenan stands out, particularly for its already commercialized nasal spray applications against numerous viral agents, comprising coronaviruses. Lectin proteins also have demonstrated extraordinary antiviral effect. Some, like cyanovirin and griffithsin, are currently regarded as main antiviral references and among the most promising molecules to fight long-fought viruses such as HIV.

The significant number of patents registered reveals the interest of some of the main economic powers for this field. The USA has led for several decades but it is now matched by China. Perhaps more surprisingly these are closely followed by Japan, a country that has some of the oldest traditions in algae exploitation. A growing record of new research and patents every year, shows the field is well alive and in expansion. Encouraging clinical trials are underway that could soon lead to algae-based solutions to difficult viruses, including HIV and coronaviruses.

There is clearly a high potential for the use of algae as unique platforms or sources to detect and develop new antivirals for a wide range of viruses for use in a diverse range of situations. Hitherto, such activities have been dominated by the desire to treat human related diseases. Yet, the potential exists for sectors such as agriculture, aquaculture and livestock production, with a high direct impact in the global economy to also benefit in the future. Whilst viruses continue to dominate the narrative of 2020, it is reassuring to know that nature has provided a bountiful source of ammunition with which to combat them, we need only look the algae for inspiration.

## Figures and Tables

**Table 1:** Relevant in vivo trials with algae antivirals.

Compound	Organism	Viruses	Model	Activity
Fucoidan	*Undaria pinnatifida*	Avian influenza A virus	Mice	Decreased viral replication, while augmenting the production of antibodies [39]
		Avian influenza A virus	Mice	Reduced viral replication, weight loss and mortality. Increased life span. Induced no viral resistance [40]
		Influenza A (H1N1) virus	Mice	Improved clinical signs of ill-health and reduced gross lung pathology [42]
	*Kjelmaniella crassifolia*	Influenza A virus	Mice	Intranasal administration markedly improved survival and decreased viral titers. Induced no viral resistance [36]
	*Fucus evanescens*	Herpes simplex virus-2	Mice	Intraperitoneal administration protected reduced lethal infection by 50% [28]
Griffithsin	*Griffithsia sp.* (Rhodophyta)	SARS-CoV (Coronaviridae)	Mice	High viral dose—100% survival compared to 30% in control group [43]
		Japanese encephalitis virus (Flaviviridae)	Mice	Lethal viral dose—100% survival compared to 0% in control group [44]
		Hepatitis C virus	Mice	Viral load below detection in infected mice [45]
		Herpes simplex virus-2	Mice	Significantly protected mice from vaginal infection (0/5 treated vs 3/5 control) [46]
		Herpes simplex virus-2/Human papilloma virus	Mice	Significantly protected mice from HSV-2 vaginal infection and HPV-16 [47]
Cyanovirin-n	*Nostoc ellipsosporum* (Cyanobacteria)	HIV-1	Macaques	Treated with topical gel were resistant to a chimera of HIV viruses [48,49]
		HIV-1	Macaques	63% reduction of HIV transmission when dosed vaginally with a *Lactobacillus* expressing CV-N [50]
Scytovirin	*Scytonema varium* (Cyanobacteria)	Ebola virus	Mice	Prevented the death of most infected mice [51]
Diterpene	*Canistrocarpus cervicornis* (Dictyotales)	Herpes simplex virus	Mice	Significantly less lesions when treated with a 2% diterpene ointment [52]

**Table 2:** Clinical trials with algae antivirals.

Compound	Organism	Intervention	Condition	Main Sponsor	Date	Outcome
Griffithsin	*Griffithsia sp.* (Rhodophyta)	Q-Griffithsin (Q-GRFT) enema	HIV Prevention	National Institute of Allergy and Infectious Diseases (USA)	2021	On-going [163]
		Griffithsin Gel	HIV Infection	Population Council	2018	Griffithsin and carrageenan in a gel formulation are safe for vaginal use for up to 14 days with potent anti-HIV activity in cell-based assays and cervical explants [162]
Carrageenan	Red seaweed (Rhodophyta)	Carraguard (PC-515)	HIV Prevention	Population Council	2007	This study did not show Carraguard’s efficacy in prevention of vaginal transmission of HIV [164]
					2004	Daily Carraguard vaginal gel use was highly acceptable in this population of HIV-infected women [165].
					2003	Vaginal use of the gel did not cause significant adverse effects in a small number of low-risk, sexually abstinent women [166]
					2002	Carraguard was not associated with more vaginal, cervical or external genital irritation than placebo and it was acceptable when used approximately 3.5 times per week, including during sex [167]
					2002	Men and women found the gel acceptable and thought that it should be made available if it is found to be safe and effective [168]
					1997	Use of 5 mL of a 2% gel formulation of n-carrageenan was not associated with significant irritation of the vaginal epithelium when administered once daily in the absence of sexual intercourse [169]
			HIV & HsV infections	Population Council	2001	Carraguard can safely be used an average of four times per week with or without sex and is acceptable to women [170]
		Coldamaris (with Carragelose)	Symptoms of common cold	Marinomed Biotechnologie GmbH (Austria)	2015	Direct local administration of carrageenan with nasal sprays reduced the duration of cold symptoms. A significant reduction of viral load in the nasal wash fluids of patients confirmed similar findings from earlier trials in children and adults [171]
				St. Anna Children’s Hospital (Austria)	2011	Administration of carrageenan nasal spray in children as well as in adults increased viral clearance and reduced relapses of symptoms [172]
		Carrashield	Human papillomavirus infection	Canadian Institute of Health Research (Canada)	2009	Trial’s interim analysis suggests that using a carrageenan-based lubricant gel can reduce the risk of genital HPV infections in women [158]
		Carvir	Human papillomavirus infection	University of Palermo (Italy)	2019	Carvir vulvovaginal microbicide gel is safe and well-tolerated [159]
		Carrageenan-based gel	Human Papillomavirus Infection	McGill University (Canada)	2020	On-going [160]
Spirulan	*Arthrospira platensis* (cyanobacteria)	Spirulan containing cream	HsV-1 infection	Leibniz Institute for Experimental Virology (Germany)	2016	Prophylactic effect against Herpes labialis was superior to that of acyclovir cream [85]
Algal Extract	*Undaria* and *Arthrospira*	Extract	HIV Infection	University of South Carolina (USA)	2008	Undaria, Arthrospira and a combination of both were non-toxic and over time may improve clinical endpoints of HIV/AIDS [173]
